# Combining Untargeted and Targeted Proteomic Strategies for Discrimination and Quantification of Cashmere Fibers

**DOI:** 10.1371/journal.pone.0147044

**Published:** 2016-01-20

**Authors:** Shanshan Li, Yong Zhang, Jihua Wang, Yunfei Yang, Chen Miao, Yufeng Guo, Zhidan Zhang, Qichen Cao, Wenqing Shui

**Affiliations:** 1 Key Laboratory of Systems Microbial Biotechnology, Tianjin Institute of Industrial Biotechnology, Chinese Academy of Sciences, Tianjin, 300308, China; 2 College of Life Sciences, Nankai University, Tianjin, 300071, China; 3 Tianjin Textile Engineering Research Institute, Tianjin, 300308, China; Pacific Northwest National Laboratory, UNITED STATES

## Abstract

Cashmere is regarded as a specialty and luxury fiber due to its scarcity and high economic value. For fiber quality assessment, it is technically very challenging to distinguish and quantify the cashmere fiber from yak or wool fibers because of their highly similar physical appearance and substantial protein sequence homology. To address this issue, we propose a workflow combining untargeted and targeted proteomics strategies for selecting, verifying and quantifying biomarkers for cashmere textile authentication. Untargeted proteomic surveys were first applied to identify 174, 157, and 156 proteins from cashmere, wool and yak fibers, respectively. After marker selection at different levels, peptides turned out to afford much higher selectivity than proteins for fiber species discrimination. Subsequently, parallel reaction monitoring (PRM) methods were developed for ten selected peptide markers. The PRM-based targeted analysis of peptide markers enabled accurate determination of fiber species and cashmere percentages in different fiber mixtures. Furthermore, collective use of these peptide makers allowed us to discriminate and quantify cashmere fibers in commercial finished fabrics that have undergone heavy chemical treatments. Cashmere proportion measurement in fabric samples using our proteomic approach was in good agreement with results from traditional light microscopy, yet our method can be more readily standardized to become an objective and robust assay for assessing authenticity of fibers and textiles. We anticipate that the proteomic strategies presented in our study could be further implicated in discovery of quality trait markers for other products containing highly homologous proteomes.

## Introduction

Animal hair fibers, such as cashmere, wool and yak, are an important source of raw materials in today's textile industry. Cashmere is the downy hair produced by goats (*Capra hircus*). It is one of the finest and softest materials used for manufacturing high quality luxury textiles. The price gap between cashmere and wool is quite significant, which can be 30-fold or even higher [1]. Yak is the fine undercoat fiber of *Bos grunniens*. Although the external fiber morphology of yak is very similar to that of cashmere, the price of yak is only a quarter that of cashmere [2]. Due to the financial interests and the increasing market demand, there is a rising trend of adulteration of cashmere with cheaper fibers, typically wool or yak, in commercial textiles. Even back to the years 1990–2003, more than two-thirds of cashmere samples analyzed were found to be adulterated [3]. For the sustainable development of textile industry, it has become imperative for cashmere producers and commercial trading partners to accurately assess the quality attributes of raw and processed products.

Currently employed methods for fiber quality assessment involve optical microscopy and scanning electron microscopy (SEM) which give information on the surface morphology, internal pigmentation and the fine structure of cuticle cells in the fiber at high resolution [4, 5]. These conventional methods are subjective and heavily depend on the skill and expertise of the operator. A large number of fibers have to be manually examined in regard to fiber diameter, crimp, color, staple strength, etc. Even though, distinguishing fine yak hair and brown cashmere is very difficult or even impossible [6]. Furthermore, the time-consuming procedure and high cost of SEM analysis has hampered its application. More recently, several other techniques have been developed to improve the objectivity and accuracy of fiber blend identification, including analysis of the external and internal lipids [7] or DNA [1, 8, 9] in fibers or developing ‘anti-cashmere’ monoclonal antibodies [10]. Nevertheless, these techniques have not been widely pursued because of concerns in method robustness and cost, especially the accuracy of results often affected by chemical treatments of fibers during textile manufacturing [11].

The major components of mammalian hair fibers are complex mixtures of proteins, yet lipids and carbohydrates are also present [12]. Approximately 90% of the fiber substance by weight is contributed by protein constituents including keratin and keratin associated proteins (KAPs) prominently [13]. Proteomic investigation of fiber composition has been mainly focused on human hair and wool fibers [14–17]. A total of 113 proteins within twenty-five keratin and KAP families were identified in human hair fibers [14, 15, 18]. Among them, 70 proteins classified into 19 keratin and KAP families were also found in wool fibers [19]. In addition, recent work from Clerens and Koehn *et al*. documented 113, 108 and 118 proteins identified in wool fibers respectively [16, 17, 20]. Apart from qualitative proteomic mapping, quantitative comparison using the iTRAQ approach uncovered an interesting correlation of wool fiber quality parameters with expression changes of specific KAPs [19, 21]. Therefore, it is conceivable that mass spectrometry analysis of species-related proteins or peptides would offer a rather promising solution to textile authentication, which was shown feasible in exploratory research by Sforza *et al* [11, 22].

In this study, we first implemented state-of-the-art proteomic techniques to profile the proteomes of wool, cashmere and yak using authentic fiber standards. Then species-specific markers were selected according to the untargeted proteomic data. A parallel reaction monitor (PRM) approach was developed for selective detection and accurate quantification of putative peptide markers. These markers further validated with blended fibers were then used to identify fiber species and quantify cashmere percentages in textile fabrics. Results from the targeted proteomic analysis were comparable with findings using traditional light microscopy, indicating the strong potential of our approach in authenticity assessment of textile products.

## Materials and Methods

### Fiber sample preparation

Six types of fiber standards compared in this study are Inner Mongolia white cashmere (C1), Inner Mongolia grey cashmere (C2), Tibetan cashmere (C3), Mian Yang Rong wool from China (W1), Australian Merino wool (W2) and Inner Mongolia yak (Y), and they were provided by Tianjin Textile Engineering Research Institute (Tianjin, China). Fiber standards of different species (5 mg each) were finely minced with scissors, washed, and proteins were extracted according to a modified method previously described [11]. In brief, ethanol was added to completely immerse the fibers. After shaking gently at room temperature for 30 min, the ethanol was filtered out with a Buchner funnel. The dichloromethane/methanol (2:1, *v/v*) solution was added to wash the fibers by shaking for 24 h before the solvent was removed by filtration. Extraction buffer (25 mM Tris-HCl, 5 M urea, 2.4 M thiourea, 5% DTT, pH 8.5) of 500 μl was added to the pretreated fibers, and left in contact with fibers for 16 h at 50°C under gentle shaking. Extracted fibers were taken out with tweezers, and the extraction solution was centrifuged under 12000g for 10 min. The supernatant was kept at -80°C and the protein concentration was determined using the Bradford assay. Two to six process replicates were prepared for each type of fiber standard, resulting in 24 samples in total. For QC sample and calibration curve preparation, fiber standards of different species were mixed at specific ratios prior to washing and protein extraction. Blend fibers drawn out from four textile fabrics were prepared in the same manner. Triplicate replicates for all QC samples and textile fabrics were prepared for IDA or PRM analysis.

### Protein digestion and dimethyl labeling

Proteins (~100 μg) in the extraction solution were precipitated by acetone at a protein-to-acetone ratio of 1:5 (v*/v*) for 2 h at -20°C, then centrifuged at 6000g for 10 min. After washing for 3 times with cold acetone, the pellet was dried under nitrogen gas and resuspended with 8 M urea and reduced with 10 mM DTT at 37°C for 2 h in a thermo mixer (Thermo Fisher Sicentific, USA). The solution was centrifuged and alkylated with 40 mM iodoacetamide at room temperature in darkness for 40 min. Additional 30 mM DTT was added to consume the excess iodoacetamide, followed by vortexing and incubation for at 37°C for 30 min. Urea was diluted with 100 mM ammonium bicarbonate to a final concentration of less than 1 M. Proteins were digested with trypsin (Promega, Madison, USA) at an enzyme-to-protein ratio of 1:100 (*w/w*) at 37°C for 2h, followed by adding fresh trypsin at 1:50 (*w/w*) and incubation at 37°C overnight. After acidification, the protein digest was desalted with C_18_-SepPak columns (Waters, Milford, USA) and lyophilized under vacuum. Equal amounts of peptides from fiber standards W1, C1 and Y were mixed and used as reference.

For triplex dimethyl labeling of peptides [23], 20 μg tryptic digest of each fiber sample was suspended in 100 μl TEAB solution (100 mM, pH 8.0). Then 4 μl of formaldehyde (CH_2_O, CD_2_O and ^13^CD_2_O, 4%, *v/v*) was added to specific samples for differential isotope labeling. The same volume of fresh 0.6 M cyanoborohydride (NaBH_3_CN) was added subsequently to the light and intermediate-labeled peptides and the labeled cyanoborohydride (NaBD_3_CN) was added to the heavy-labeled peptides, and incubated for 1 h at room temperature. Then 20 μl of 1% ammonium solution was added and vortexed for 10 min. Finally, 8 μl of 5% formic acid was added to quench the reaction. In each triplex labeling set, the peptides of the reference and two process replicates of one fiber sample were labeled with light, intermediate and heavy isotope tags respectively. Triplex-labeled peptides from different samples were mixed at equal amount, and desalted prior to nanoLC-MS/MS analysis.

### NanoLC-MS/MS analysis

The nanoLC-MS/MS analysis was conducted on an Eksigent NanoLC connected to TripleTOF^TM^ 5600 mass spectrometer (Applied Biosystem, USA) with a nano-electrospray ionization source. The sample (~1 μg peptides) dissolved in solvent A (0.1% formic acid, 2% acetonitrile) was loaded onto a trap column (10 mm×100 μm, 5 μm C18 resin) and separated on an analytical column (100 mm×75 μm) in-house packed with C18-AQ 3 μm C18 resin (Dr. Maisch, GmbH, Germany), using a gradient of 5–36% solvent B (0.1% formic acid, 98% acetonitrile) over 82 min at flow rate of 300 nl/min. The mass spectrometer acquisition method was set to the following parameters: 2300 V ion spray voltage, 30 psi curtain gas, 10 psi ion source gas, and 150°C interface heater temperature.

For untargeted proteomic analysis, the mass spectrometer operated in the information-dependent acquisition (IDA) mode. The survey scan mass range was 350–1500 *m/z*, following a “top 40” MS/MS scan with the mass range of 100–1500 m/z. The dynamic exclusion time was set at 22 sec. For targeted peptide analysis, the mass spectrometer was programmed in the “product ion” mode in TripleTOF^TM^ 5600 system. The precursor isolation width was set to unit, and the fragment ion scan range was set to 350–2000 *m/z* with an accumulation time of 50 ms. The PRM methods for targeted peptides were developed based on the IDA results. All precursor ions of targeted peptides with varying charge states detected in the IDA mode were built into the acquisition list. Collision energy was automatically optimized for each peptide precursor according to its *m/z* value to acquire high-quality MSMS spectra. The top 3 to 6 product ions by intensity constructed transitions for individual peptide precursors. All the transitions were validated using the mProphet algorithm in Skyline advanced peak picking model [24, 25]. The raw MS data has been deposited to the ProteomeXchange Consortium via the PRIDE partner repository with the dataset identifier PXD003107 (Username: reviewer42019@ebi.ac.uk; Password: WA3l3UhH).

### IDA data processing

For qualitative profiling purpose, IDA data were processed using Mascot search engine (v2.5.1, Matrix Science, London, UK). The Uniprot databases of wool (Ovis aries, Nov-2013, 4221 entries), cashmere (Capra hircus, Nov-2013, 2004 entries) and yak (Bos mutus grunniens & Bos grunniens mutus, Nov-2013, 18907 entries) were combined to build a collective fiber proteome database. Precursor ion mass tolerance was set to 20 ppm and fragment ion tolerance was 0.05 Da. Trypsin was set as the specific enzyme and two missed cleavages were allowed. Carbamidomethyl was set as fixed modification, dimethyl labeling and methionine oxidation were set as variable modifications. For protein identification, the Mascot cutoff false discovery rates (FDR) were set at 5%, and ion score cutoff was 20. A decoy database was searched to ensure the actual FDR with the aforementioned criteria was below 1% for both protein and peptide identification.

ProteinPilot^TM^ (v4.5, AB SCIEX, Foster City, CA) equipped with Paragon Algorithm was employed to obtain the relative ratios of proteins identified in each fiber sample against reference. The software performs automatic recalibration such that typical mass errors for MS and MS/MS data were below 20 ppm. The same collective fiber proteome database was searched, with trypsin specified digestion, and variable cysteine alkylation with iodoacetamide. ProteinPilot automatically clusters the identified proteins into protein groups sharing common peptides. Only protein identifications with >99% confidence were retained, resulting in FDR <1% as calculated by a decoy database search. For quantification analysis, dimethyl labeling was specified to be the quantification method. Only peptides of unique sequences (not shared with other proteins) and free of miscleavages or variable modifications contributed to protein ratio calculation. Protein ratios in specific fiber samples *vs* reference were derived from at least two unique quantified peptides, and they were normalized using the median protein ratio.

### Multivariate analysis

Protein ratios for different fiber samples were reformed into an Excel matrix which was then imported into SIMCA software (v14.0, Umetrics AB, Sweden) for multivariate statistical analysis. Partial least squares-discriminate analysis (PLS-DA) was employed to identify biochemical patterns. The value of variable importance in the projection (VIP) in PLS-DA model and the result of ANOVA tests between different groups were combined to determine biomarker candidates. Proteins with VIP >1.0 and ANOVA *p*-value <0.01 were regarded as putative markers for fiber species specification.

### PRM data processing and quantification of cashmere proportion

The PRM data were processed using Skyline (v2.6.0) with detailed settings specified by the software instruction [26]. Only b- or y- fragment ions with *m/z* values greater than the precursor were selected to build the elution profile for peptide quantitation. All extracted ion chromatograms (XICs) of selected fragments were manually inspected and adjusted to ensure proper peak picking and peak integration. The XIC intensity ratio of targeted peptides from a given fiber sample *vs* reference was defined as the normalized peptide responses. A calibration curve was constructed based on normalized peptide responses measured from a set of C1 and W1 mixtures with varying C1 percentages (5%-100%, at least 7 data points). The cashmere proportion in fiber mixtures and finished fabrics were determined using the calibration curve from three process replicates.

### Scanning electron microscopy of fiber samples

The fiber standards and fibers from finished fabrics were analyzed using a scanning electron microscope (Hitachi SU8010, Japan), to acquire multiple high-resolution images of the external morphology of the cuticle cells in fibers. The fibers were placed in the test chamber of SEM using snippets, and representative digital images were captured at x2200 magnification to evaluate the structural characteristics of specific fibers.

## Results

### Proteomic profiling of fiber standards from different species

**Fig 1:**
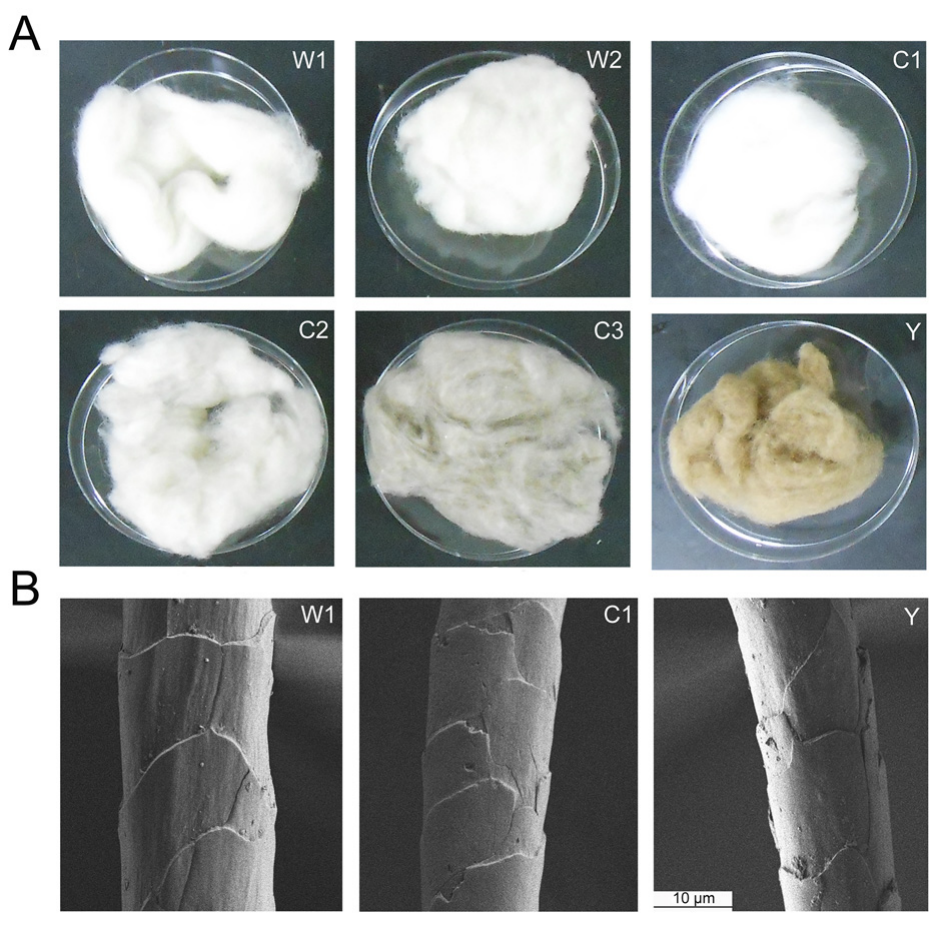
Animal hair fiber standards involved in our proteomic study. (A) Appearance inspection of different fiber standards including two breeds of wool (W1, W2), three breeds of cashmere (C1, C2, C3) and one breed of yak (Y) as specified in Experimental Section. (B) Scanning electron microscopy (SEM) analysis of the fine morphology of different fibers.

**Fig 2:**
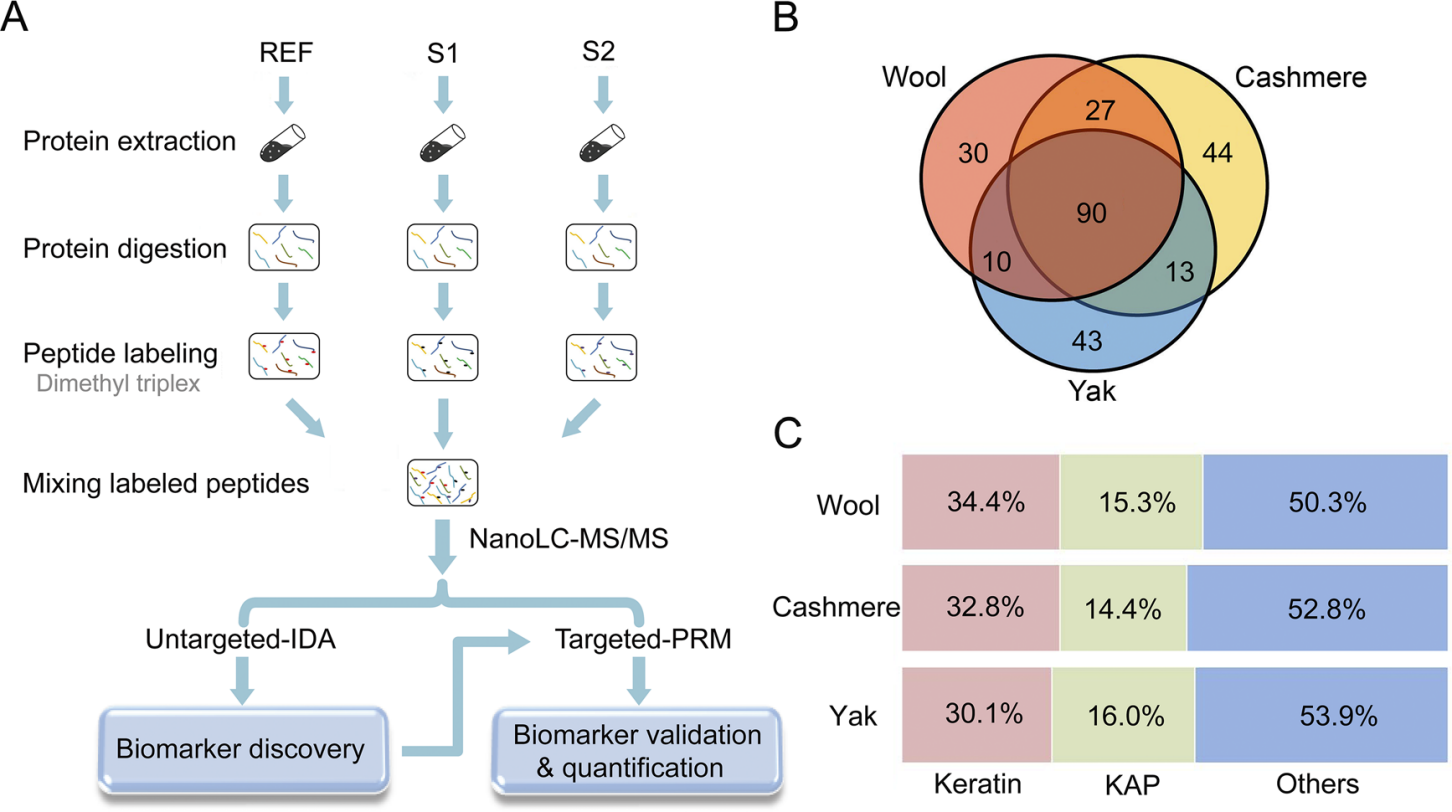
The quantitative proteomic strategy for fiber marker discovery and fiber proteome profiling results. (A) The schematic workflow of marker discovery and validation with combined untargeted and targeted proteomic strategies. Two replicates of a specific fiber sample (S1 and S2) were labeled with intermediate and heavy dimethyl tags, a fiber mixture from three species with a light dimethyl label served as reference (REF). The mixture of S1, S2 and REF was analyzed by nanoLC-MS/MS in IDA mode for fiber proteome profiling and marker discovery. Then peptide markers were validated and used for fiber quantification with the parallel reaction monitoring (PRM) approach. (B) Numbers of protein identification in cashmere, wool and yak fiber samples. The result shows large overlap of the fiber proteome among three species. (C) Percentage of keratin and KAPs identified in three types of fibers. Detailed GO classification for each identified protein is shown in S1 Table.

Six types of fiber standards from three breeds of cashmere, two breeds of wool and one breed of yak were characterized in this study (Fig 1A). The breeds selected here represent prevalent types of cashmere present in textiles in Chinese markets, and the wool and yak breeds are typically found blended with cashmere fibers for adulteration. Notably, SEM imaging of fiber morphology did not show apparent differences between cashmere and wool or yak, indicating challenges in fiber species differentiation solely based on fine structural analysis (Fig 1B). To address this issue, we devised a proteomic workflow combining untargeted and targeted analysis to discover species-specific fiber markers which can be used for determination of fiber composition in commercial textile products. As shown in Fig 2A, a triplex dimethyl-labeling approach was employed for protein identification and quantification in authentic fiber standards [23]. A mixture of cashmere, light dimethyl-labeled wool and yak fibers served as reference and was spiked into replicates of specific fiber samples with intermediate and heavy dimethyl labels. Therefore, the protein ratios in each fiber replicate *vs* reference correlated to protein abundances in different fiber samples. We first applied an untargeted proteomic strategy to profile the proteomes of different fiber standards and identify protein or peptide markers that can distinguish fiber species. These putative markers were then validated with a PRM-based targeted approach, and effective markers were used to determine cashmere composition in fiber mixtures and textile fabrics.

After combining identification results from 2~6 replicates of each fiber standard, we reported 157 non-redundant proteins from two breeds of wool, 174 proteins from three breeds of cashmere and 156 proteins from yak respectively (protein summaries in S1 Table). Ninety proteins are shared by three species whereas 44, 30 and 43 proteins are unique in cashmere, wool and yak fibers, respectively (Fig 2B). The fiber proteomes profiled in our study consist of keratins and keratin-associated proteins (KAPs) in a significant fraction, which are known to be the predominant structural components of the intermediate filament in fibers [13] (Fig 2C). Owing to the depth of our proteomic analysis, we also identified a variety of proteins engaged in cell adhesion, signal transduction, metabolic process, transport & transcription, etc. in the fiber extracts. GO classification of identified proteins in different fiber species are shown in S1 Table.

### Selection of candidate markers for discrimination of fiber standards

**Fig 3:**
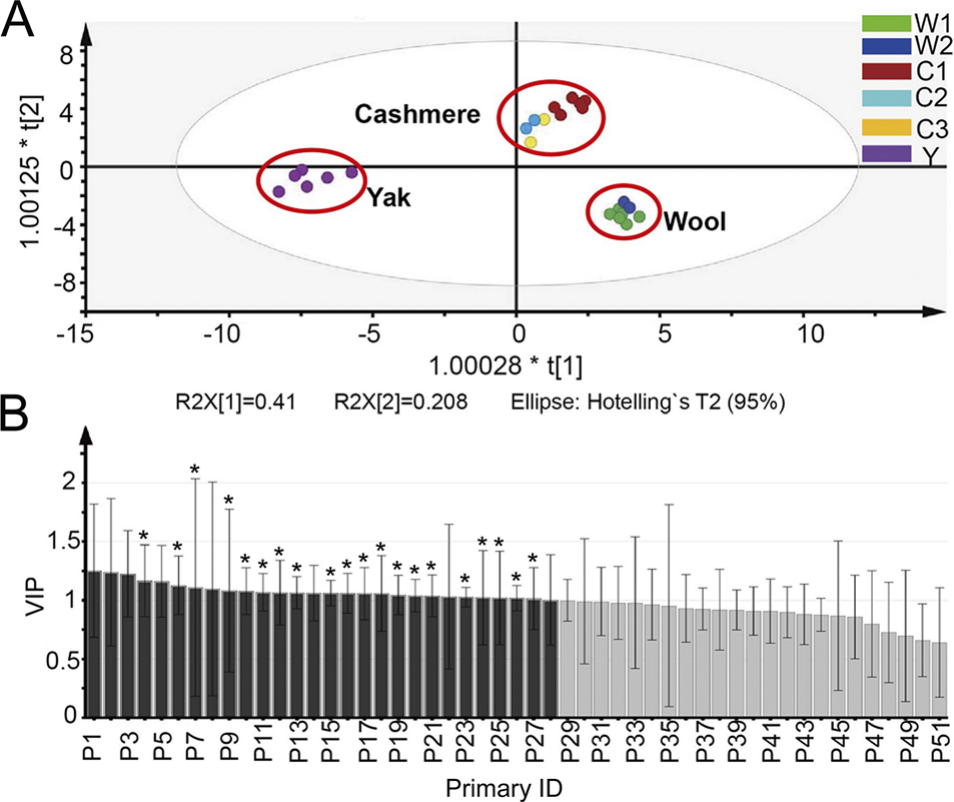
The PLS-DA analysis performed on quantified proteins from fiber standards. (A) The PLS-DA scores plot shows good separation of samples from different species, and samples from different breads of the same species are clustered together. (B) The variable influence on projection (VIP) plot shows that protein P1-P28 (with VIP value > 1) make most contribution to the separation of three groups in (A). Twenty out of the twenty-eight proteins also make significant discrimination in the ANOVA test (*p*<0.01) and they are marked with asterisks.

In the IDA-based untargeted proteomic experiment, we simultaneously obtained protein quantification data from fiber standards using the dimethyl labeling technique (S2 Table). Sufficient reproducibility of quantification was implied by Pearson correlation of protein ratios >0.8 and average RSD <30% across multiple process replicates. Partial least-squares discriminant analysis (PLS-DA) widely employed for clinical biomarker discovery [27] was then conducted to develop a compliance measure of species origin based on the most discriminative proteins detected in fiber standards. The pretreated data matrix from proteins quantified in all six fiber standards was used to develop the PLS-DA model, with the origin patterns given as class information. The result exhibited a well-segregated, closely clustered pattern among various groups in the PLS-DA score plot (Fig 3A). Replicates of the same sample are all clustered together, again suggesting good reproducibility of proteomic quantification. More importantly, cashmere and wool fibers of different breeds are clustered into the same group, suggesting they have similar molecular signatures that can be easily distinguished between each other and from yak.

To select candidate protein markers with the most influence in discriminating fiber species, we applied combined cut-offs of VIP >1 in the PLS-DA model and *p* value in an ANOVA test <0.01. Applying the double criteria, twenty significant discriminating proteins such as keratin associated protein 13.1 (KAP13-1), hair acidic keratin 1 (K31), keratin type II cytoskeletal 1 (fragment) (K1) and keratin, type II cytoskeletal 7 (K7) were discovered (Fig 3B). To verify these candidate markers in determination of fiber composition, we prepared fiber standard mixtures containing various proportions of cashmere and analyzed them with the untargeted proteomic approach. As shown in S1 Fig, none of the candidate markers presented a consistent increase (or decrease) of their quantitative values with the increasing cashmere amount in the mixtures. As the PLS-DA model-derived protein markers failed to predict the quantitative changes of cashmere in mixed samples, they were not pursued further for quality assessment of cashmere products.

**Fig 4:**
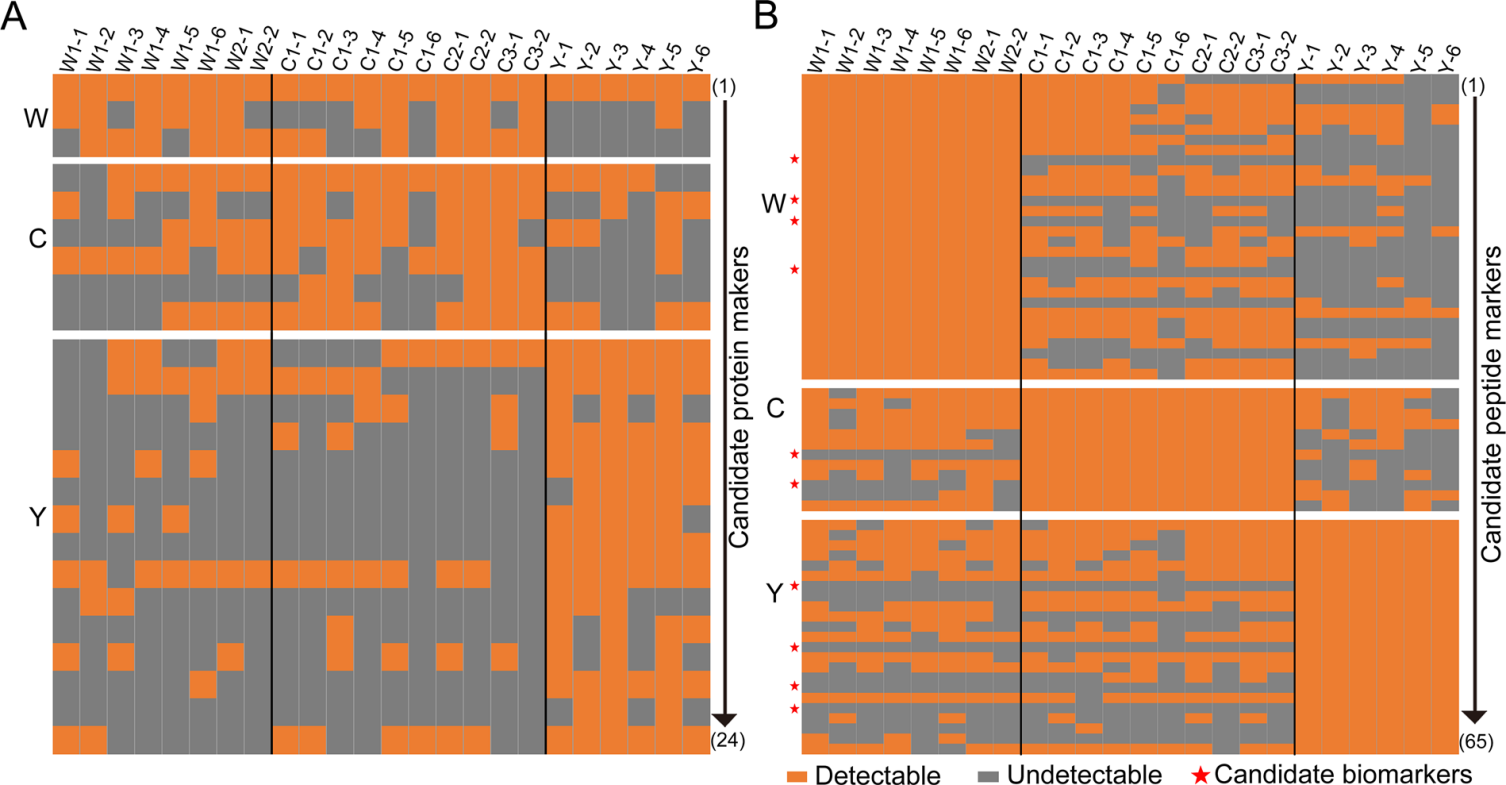
Evaluation of candidate markers in species identification of fiber standards. (A) Evaluation of 24 candidate protein markers selected for fiber identification. None of them show satisfactory specificity and sensitivity across all fiber samples. (B) Evaluation of 65 candidate peptide markers selected for fiber qualification. Ten peptides (marked with a red star) show sufficient specificity and sensitivity across all fiber samples. Each line represents identification results of a specific protein/peptide across all samples. Fiber sample annotation is the same as described in Fig 1, and the appending number refers to the number of replicate of this sample.

Next, we evaluated the discrimination power of unique proteins identified in three fiber species (Fig 2B). Proteins pooled from replicates of the same sample and commonly detected from different breeds of the same species were first grouped, and then the ones specific to only one species were selected as candidate markers. Altogether, 24 proteins were picked, including 6 from cashmere, 3 from wool and 15 from yak, and they were first verified in all 24 fiber standards prepared separately and analyzed with the IDA method. Protein markers of high specificity are expected to be detected only in samples of the same origin and absent in samples from the other two species. However, for the six candidate cashmere markers, some of them were identified in wool or yak samples while others were not consistently identified in all cashmere samples (Fig 4A). The candidate markers for wool and yak were also unable to accurately differentiate all fiber samples (Fig 4A). Therefore, at this stage, our efforts in search of protein markers failed, which was attributed to the substantial homology of keratin and KAPs in different fiber proteomes that led to overlapping protein identifications across species.

Finally we resorted to the peptide collections in different fiber samples to search for effective markers. Similar to protein-level data processing, we first grouped all peptides identified in multiple breeds of the same species, and then the peptides unique to one species were selected as candidate markers. When verifying these peptide markers with the aforementioned set of fiber samples, 10 out of 65 candidates were positive in all samples of the same origin and negative in samples from the other two species, demonstrating adequate specificity and sensitivity for discrimination of fiber standards (Fig 4B). These ten peptide markers include 2 peptides from cashmere, 4 from wool and 4 from yak.

### Identification and quantification of fiber mixtures through targeted analysis of peptide markers

**Fig 5:**
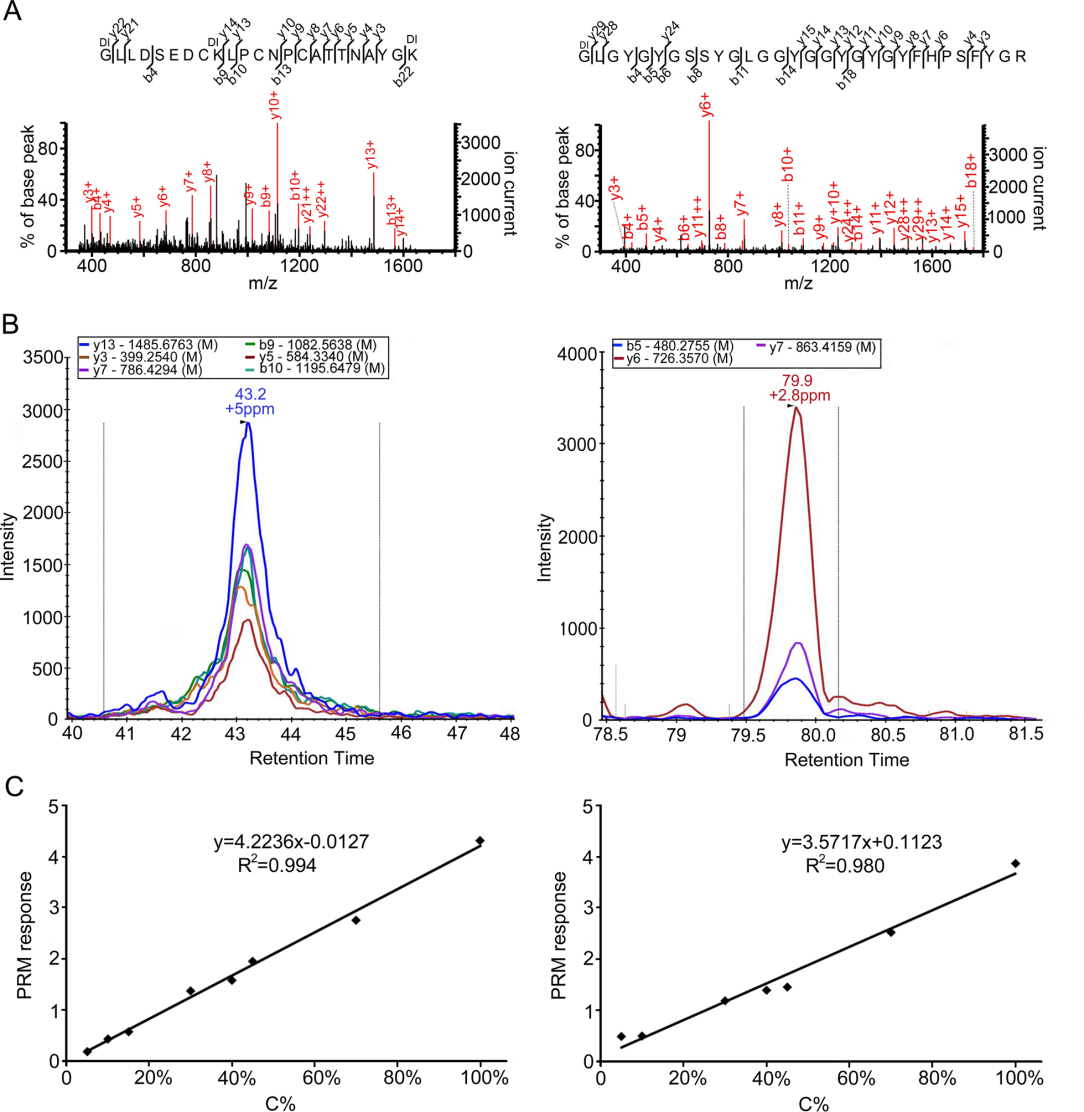
Two peptide markers discovered in our study for discrimination and quantification of cashmere fibers. (A) The MS/MS spectra of the two cashmere marker peptides obtained from cashmere-wool mixtures. (B) The PRM transitions of cashmere markers extracted by Skyline software. (C) The standard curves established for two cashmere marker peptides using PRM analysis. W1 and C1 mixtures were prepared with varying C1 percentages.

Putative peptide markers were then evaluated using QC samples of mixed fibers with known proportions of fiber components. To increase the sensitivity and selectivity of marker detection, we performed targeted peptide analysis using the parallel reaction monitoring (PRM) approach [28]. In PRM experiments, all product ions of targeted peptide precursors are recorded by high-resolution mass spectrometry (HRMS), and suitable products are selected to establish MRM-like transitions for precursor detection and quantification. The main advantage of PRM is to separate interferences from the true signals by HRMS, thus significantly enhancing the selectivity of the method compared to the conventional MRM approach [29]. The MSMS spectra and XICs of selected product ions in PRM experiments for two cashmere peptide markers are shown in Fig 5A and 5B. The PRM transitions and sequences of ten peptide markers are listed in S3 Table. Using this targeted PRM approach, all peptide markers were correctly detected in five different QC samples without false positives or negatives, which verified the use of peptide markers in fiber species discrimination (S4 Table).

The PRM approach was also employed to quantify the proportion of cashmere in QC samples. Calibration curves for the two cashmere peptide markers were first constructed using W1 and C1 mixtures with varying C1 percentages. Both peptides exhibited excellent linear responses over the range of 5% to 100% C1, with the regression factor (R^2^) of 0.994 and 0.980 (Fig 5C). The low limit of quantification (LLOQ) is estimated to be 5% cashmere for peptide C1 and 10% for peptide C2, which is sufficient for quality assessment of commercial textiles. Cashmere percentages determined for three QCs containing 20%, 33% and 60% of cashmere all showed relative errors <15% and CVs <10%, implying high accuracy and precision of our approach in quantification of fiber constituents (Table 1). Taken together, the PRM approach demonstrated adequate sensitivity and accuracy for identification and quantification of fiber mixtures.

**Table 1:** Cashmere percentages in QC samples determined using two peptide markers.

Peptide markers	Sample^*a*^	Theo. C%	Meas. C% Average	Meas. C% CV	Relative error (%)
C-1	QC1	60%	65%	8.2%	8.3%
	QC3	20%	19%	1.5%	5.0%
	QC4	33%	29%	5.5%	12.1%
C-2	QC1	60%	66%	6.0%	10.0%
	QC3	20%	18%	2.3%	10.0%
	QC4	33%	32%	5.8%	3.0%

^*a*^QC1, a mixture of W1 and C1 (2:3, *w/w*); QC3, a mixture of W1 and C1 (4:1, *w/w*); QC4, a mixture of W1, C1 and Y (1:1:1, *w/w/w*).

### Determination of cashmere proportions in textile fabrics

**Fig 6:**
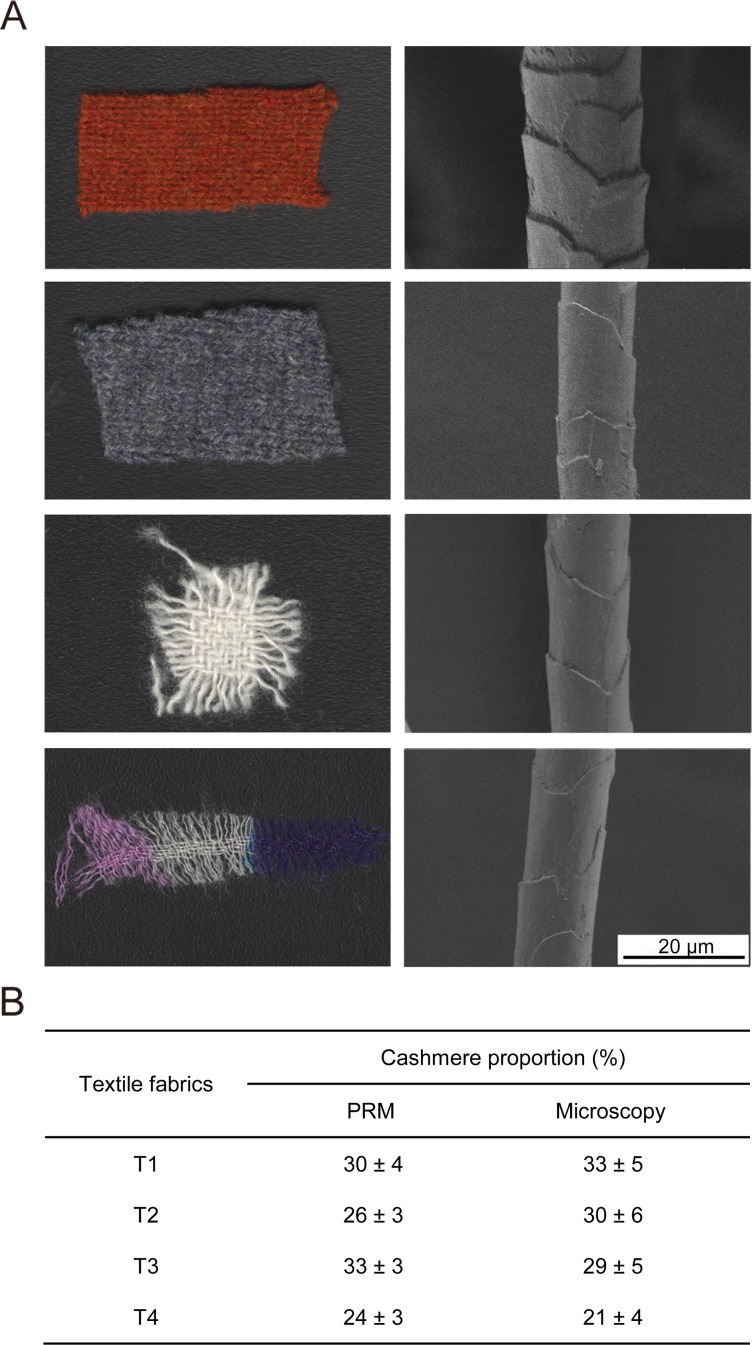
Determination of cashmere proportions in textile fabrics. (A) Four fabrics from commercial textiles analyzed in this work. On the left are the appearance of fabric samples and on the right is the SEM image of a single fiber from each fabric. (B) Quantification results of cashmere proportions in the four fabrics using the PRM approach developed in this study and the traditional light microscopy analysis.

We then used the peptide markers to determine fiber composition in four textile fabrics from commercial products (T1-T4). The fibers in these finished fabrics have been chemically treated and dyed in different color (Fig 6A). Damage of fiber morphology due to chemical treatment made it rather difficult to discriminate fiber species by SEM analysis (Fig 6B). We first relied on 10 peptide markers unique to different species for fiber identification in the fabric. Two cashmere markers as well as 2 or 3 wool markers were detected in the protein extracts of four fabrics by PRM analysis, and none of yak markers were identified in any fabric samples (S4 Table). Based on these identification data, we concluded that all four fabrics are blend of cashmere and wool fibers. The fact that not all wool markers were detected in fabric samples was presumably a result of intense chemical processing of fibers which may affect protein extraction and digestion efficiency.

PRM analysis of cashmere marker peptides allowed us to determine cashmere percentages in the textile fabrics using calibration curves prepared earlier (Fig 5C). Quantification results using either peptide marker are summarized in Fig 6B. Cashmere percentages determined using our targeted approach are in good agreement with results from traditional light microscopy analysis (relative deviation <15%) which also concluded that none of the fabric samples contained yak fibers. But light microscopy requires extensive experiences of analysts and results could vary widely from person to person. Thus our study has strong implications for authenticity assessment of textile products using proteomic strategies.

## Discussion

In the present study, we combined untargeted and targeted proteomic strategies for comprehensive profiling of several animal hair fiber proteomes and discovering effective markers for quality assessment of fibers and textiles. Compared to reported wool proteomes which mainly cover 108–118 proteins [16, 17, 20], our study provides the largest fiber proteome repository for wool (157 proteins), cashmere (174 proteins) and yak (156 proteins) owing to extensive sample analysis by high-resolution mass spectrometry.

Although the quantification results at the protein level enabled distinct separation of different fiber species in the PLS-DA model, we were unable to find any protein markers that can discriminate fiber origin. The considerable protein sequence homology and the intrinsic limitation of bottom-up proteomics are speculated to be the main reason for the unsuccessful search of protein markers. Goat (Capra hircus), Sheep (Ovis aries) and Poephagus grunniens (Bos mutus grunniens) that separately produce cashmere, wool and yak have a very high affinity in evolution, and they all belong to the Bovidae of Cetartiodactyla in Taxonomy. Among the proteins identified in our proteomic surveys of three fiber species, more than 54.5 percent are commonly detected in two or three species (Fig 2B). In addition to highly similar proteome profiles between species, keratin and KAPs, which are the primary protein components of the hair fiber, have high degree of sequence homology within each family. The homology of type I keratin we identified in cashmere can reach 94% and type II keratin can reach 82%. Due to the nature of bottom-up proteomics, a large number of overlapping peptides are detected in homologous proteins, causing ambiguous protein identification. Furthermore, extensive amino acid polymorphism and various post-translational modifications are found in keratin and KAP family members across different breeds and origins [30–33], which could lower sequence coverage for fiber protein identification. Taken together, discovery of distinctive fiber protein markers becomes quite challenging with our current proteomic approach. By contrast, we anticipate that use of top-down proteomics technology [34, 35], would resolve this problem by characterization of diverse protein isoforms and sequence variants in the intact protein dimension, thus increasing the chance of finding species-unique protein markers.

Our study identified a set of peptide markers that achieved high selectivity and accuracy in both identification and quantification of fiber composition in standard mixtures and commercial textile fabrics. Targeted PRM analysis of peptide markers holds great promise of becoming an objective and robust assay for authentication of expensive textile products. This new approach is expected to overcome the shortcoming of traditional fiber morphology evaluation using microscopy which heavily depends on the analyst’s experience and offers less reproducible results. However, we noticed that some of the wool peptide markers found in our study were not as constantly detected in finished fabrics as in fiber standards (S4 Table). For textile products undergoing chemical treatment such as bleaching, depigmentation and dying, we need to improve the efficiency of protein extraction, digestion and peptide release to assure marker identification. It is noteworthy that keratin and KAPs in fiber proteomes have high content of cysteine and relative low content of trypsin cleavable residues. Koehn *et al*. described combined use of trypsin and chemical cleavage by NTCB to improve the sequence coverage and confidence of keratin and KAP identification from wool [17, 20]. Moreover, Wilhelm *et al*. reported combining trypsin with chymotrypsin to largely improve identification of KAPs from human tissues [36]. We also investigated the effectiveness of protein cleavage with trypsin-NTCB or trypsin-chymotrypsin in fiber samples, and found an increased number of peptides unique to fiber species (data not shown). However, the reproducibility of peptide mapping was reduced with these combined cleavage reagents in our initial test. Therefore, we will continue to optimize sample pretreatment and enzymatic hydrolysis in the future work so as to further enhance the robustness and applicability of our assay.

## Conclusions

In this study, a combined proteomic workflow for identification and quantification of cashmere in blend fibers and textiles has been developed. Totally, 174, 157 and 156 proteins are identified from multiple breeds of cashmere, wool and yak, respectively. According to the quantitative proteomic data, fibers of different breeds but from the same species are distinctively clustered in the PLS-DA analysis. Although search of candidate markers fail at the protein level, a set of peptide markers distinguishing different fiber species are selected and validated in our study.

PRM methods developed for detection and quantification of putative peptide markers exhibit high sensitivity and accuracy in fiber quality assessment. Fiber species in blend samples and textile fabrics are correctly identified by collective use of 10 peptide markers, and cashmere percentages are also accurately determined using two cashmere markers.

In conclusion, the proteomic strategies presented in our work constitute an objective and practical workflow for discovery of fiber quality trait markers and determination of the exact composition of blended animal hair fibers. Similar strategies can be employed for assessing authenticity of textiles containing specific types of fibers beyond the scope of this study.

## Supporting Information

S1 Fig Quantitative changes of PLS-DA-derived candidate protein markers in different fiber standard mixtures (TIF)

S1 Table Summary of protein identification and GO classification in three species of fiber standards (a separate Excel file). (XLSX)

S2 Table Dimethyl labeling-derived quantification results for proteins identified in specific replicates of fiber samples (a separate Excel file). (XLSX)

S3 Table PRM transition list for targeted analysis of fiber marker peptides. (DOC)

S4 Table PRM identification results of 10 peptide markers in QC samples and textile fabrics. (DOC)
