# Supplementary figures and images for: Combining Untargeted and Targeted Proteomic Strategies for Discrimination and Quantification of Cashmere Fibers
Source: PLoS One. 2016 Jan 20;11(1):e0147044. doi: 10.1371/journal.pone.0147044 (PMC4720366; doi:10.1371/journal.pone.0147044)

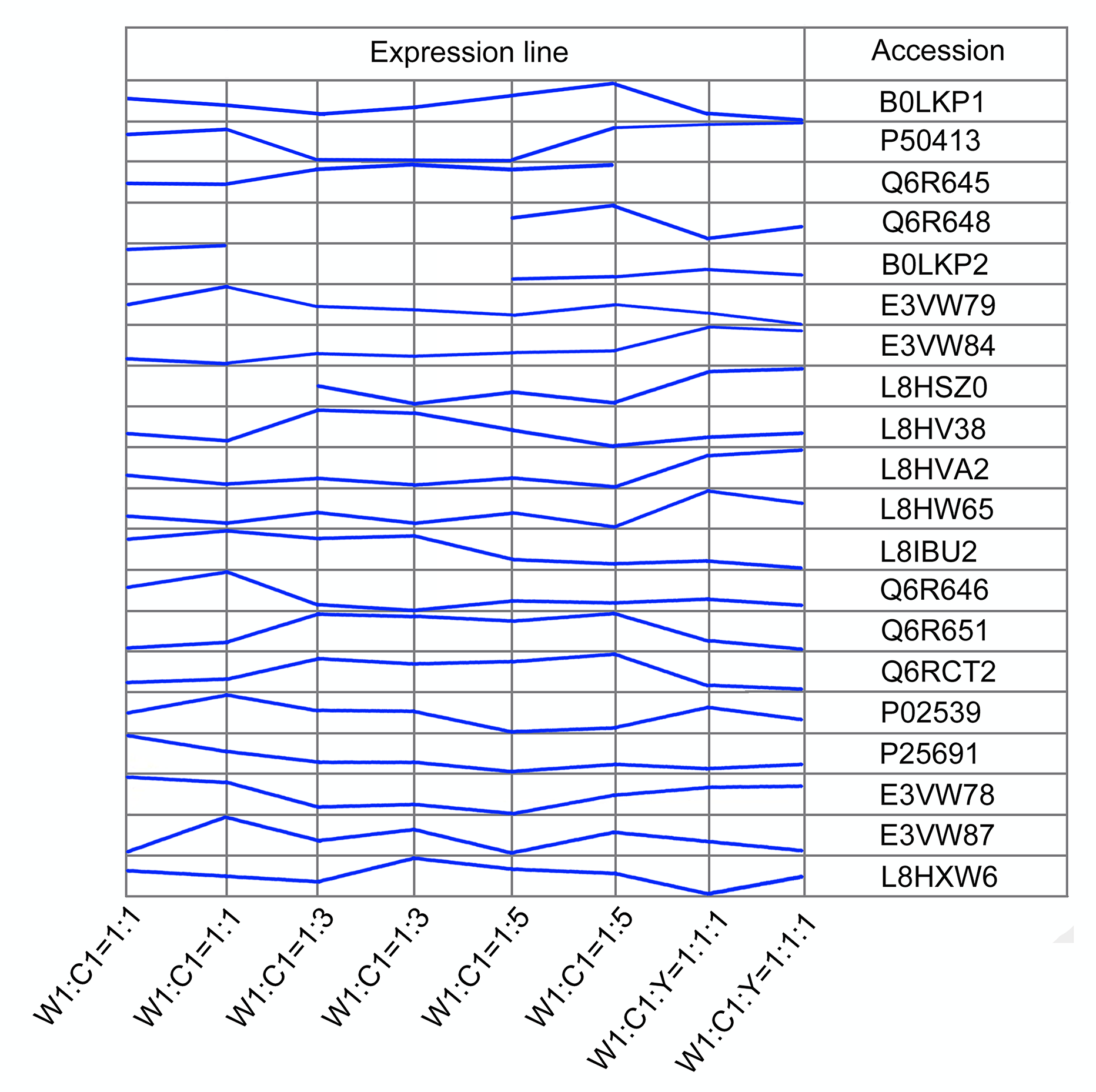

Supplement: S1 Fig — (TIF) [file pone.0147044.s001.tif]
