# Supplementary material for: Combining Untargeted and Targeted Proteomic Strategies for Discrimination and Quantification of Cashmere Fibers
Source: PLoS One. 2016 Jan 20;11(1):e0147044. doi: 10.1371/journal.pone.0147044 (PMC4720366; doi:10.1371/journal.pone.0147044)
Supplement: S3 Table — (DOC) [file pone.0147044.s004.doc]

**Supporting Information for Manuscript**

**S3 Table. PRM transition list for targeted analysis of fiber marker peptides**

| **Modified peptide** | | **Sequence** | **Charge state** | **Precursor ion (m/z)** | | **Dimethyl labeling type** | **Collision energy (V)** | | **Fragment ion (m/z)** | | **Fragment type** |
| --- | --- | --- | --- | --- | --- | --- | --- | --- | --- | --- | --- |
| **Wool** | |  |  |  |  | |  |  | |  | |
| (DI)VESLK(DI)EELIC(CAM)LK(DI) | | VESLKEELICLK | 2+ | 772.9520 | | Light | 43 | | 1417.7971 | | y11 |
|  | |  |  |  | |  |  | | 1288.7545 | | y10 |
|  | |  |  |  | |  |  | | 932.5121 | | y7 |
|  | |  |  |  | |  |  | | 871.4771 | | b7 |
|  | |  |  |  | |  |  | | 984.5612 | | b8 |
|  | |  |  | 778.9897 | | Intermediate | 43 | | 1425.8473 | | y11 |
|  | |  |  |  | |  |  | | 1296.8047 | | y10 |
|  | |  |  |  | |  |  | | 936.5372 | | y7 |
|  | |  |  |  | |  |  | | 879.5273 | | b7 |
|  | |  |  |  | |  |  | | 992.6114 | | b8 |
|  | |  |  | 785.0186 | | Heavy | 43 | | 1433.8858 | | y11 |
|  | |  |  |  | |  |  | | 1304.8432 | | y10 |
|  | |  |  |  | |  |  | | 940.5565 | | y7 |
|  | |  |  |  | |  |  | | 887.5659 | | b7 |
|  | |  |  |  | |  |  | | 1000.6499 | | b8 |
| (DI)QIASGPVATGGSITVLAPDSC(CAM)QPR | | QIASGPVATGGSITVLAPDSCQPR | 3+ | 804.0847 | | Light | 43 | | 485.2718 | | b5 |
|  |  |  |  |  | |  |  | | 681.930 | | b7 |
|  | |  |  |  | |  |  | | 752.4301 | | b8 |
|  | |  |  |  | |  |  | | 1243.6099 | | y11 |
|  | |  |  |  | |  |  | | 1043.4939 | | y9 |
|  | |  |  |  | |  |  | | 930.4098 | | y8 |
|  | |  |  |  | |  |  | | 859.3727 | | y7 |
|  | |  |  |  | |  |  | | 647.2930 | | y5 |
|  | |  |  | 805.4264 | | Intermediate | 43 | | 489.2969 | | b5 |
|  | |  |  |  | |  |  | | 685.4181 | | b7 |
|  | |  |  |  | |  |  | | 756.4552 | | b8 |
|  | |  |  |  | |  |  | | 1243.6099 | | y11 |
|  | |  |  |  | |  |  | | 1043.4939 | | y9 |
|  | |  |  |  | |  |  | | 930.4098 | | y8 |
|  | |  |  |  | |  |  | | 859.3727 | | y7 |
|  | |  |  |  | |  |  | | 647.2930 | | y5 |
|  | |  |  | 806.7661 | | Heavy | 43 | | 493.3162 | | b5 |
|  | |  |  |  | |  |  | | 689.4374 | | b7 |
|  | |  |  |  | |  |  | | 760.4745 | | b8 |
|  | |  |  |  | |  |  | | 1243.6099 | | y11 |
|  | |  |  |  | |  |  | | 1043.4939 | | y9 |
|  | |  |  |  | |  |  | | 930.4098 | | y8 |
|  | |  |  |  | |  |  | | 859.3727 | | y7 |
|  | |  |  |  | |  |  | | 647.2930 | | y5 |
| (DI)GLLDSEDTK(DI) | | GLLDSEDTK | 2+ | 517.2742 | | Light | 27 | | 758.3567 | | b7 |
|  | |  |  |  | |  |  | | 948.4884 | | y8 |
|  | |  |  |  | |  |  | | 835.4044 | | y7 |
|  | |  |  |  | |  |  | | 722.3203 | | y6 |
|  | |  |  |  | |  |  | | 607.2933 | | y5 |
|  | |  |  | 521.2993 | | Intermediate | 27 | | 762.3818 | | b7 |
|  | |  |  |  | |  |  | | 952.5135 | | y8 |
|  | |  |  |  | |  |  | | 839.4295 | | y7 |
|  | |  |  |  | |  |  | | 726.3454 | | y6 |
|  | |  |  |  | |  |  | | 611.3185 | | y5 |
|  | |  |  | 525.3186 | | Heavy | 27 | | 766.4010 | | b7 |
|  | |  |  |  | |  |  | | 956.5328 | | y8 |
|  | |  |  |  | |  |  | | 843.4487 | | y7 |
|  | |  |  |  | |  |  | | 730.3647 | | y6 |
|  | |  |  |  | |  |  | | 615.3377 | | y5 |
| (DI)LC(CAM)C(CAM)SVPTSPATTIC(CAM) SSDK(DI) | | LCCSVPTSPATTICSSDK | 2+ | 1020.4760 | | Light | 54 | | 549.2160 | | b4 |
|  | |  |  |  | |  |  | | 648.2844 | | b5 |
|  | |  |  |  | |  |  | | 1392.6675 | | y13 |
|  | |  |  |  | |  |  | | 1107.5351 | | y10 |
|  | |  |  | 1024.5011 | | Intermediate | 54 | | 553.2411 | | b4 |
|  | |  |  |  | |  |  | | 652.3095 | | b5 |
|  | |  |  |  | |  |  | | 1396.6926 | | y13 |
|  | |  |  |  | |  |  | | 1111.5602 | | y10 |
|  | |  |  | 1028.5203 | | Heavy | 54 | | 557.2603 | | b4 |
|  | |  |  |  | |  |  | | 656.3287 | | b5 |
|  | |  |  |  | |  |  | | 1400.7119 | | y13 |
|  | |  |  |  | |  |  | | 1115.5794 | | y10 |
|  | |  | 3+ | 680.6531 | | Light | 37 | | 1107.5351 | | y10 |
|  | |  |  |  | |  |  | | 1010.4823 | | y9 |
|  | |  |  |  | |  |  | | 939.4452 | | y8 |
|  | |  |  |  | |  |  | | 838.3975 | | y7 |
|  | |  |  |  | |  |  | | 737.3498 | | y6 |
|  | |  |  |  | |  |  | | 624.2658 | | y5 |
|  | |  |  |  | |  |  | | 464.2351 | | y4 |
|  | |  |  | 683.3365 | | Intermediate | 37 | | 1111.5602 | | y10 |
|  | |  |  |  | |  |  | | 1014.5074 | | y9 |
|  | |  |  |  | |  |  | | 943.4703 | | y8 |
|  | |  |  |  | |  |  | | 842.4226 | | y7 |
|  | |  |  |  | |  |  | | 741.3749 | | y6 |
|  | |  |  |  | |  |  | | 628.2909 | | y5 |
|  | |  |  |  | |  |  | | 468.2602 | | y4 |
|  | |  |  | 686.0160 | | Heavy | 37 | | 1115.5794 | | y10 |
|  | |  |  |  | |  |  | | 1018.5267 | | y9 |
|  | |  |  |  | |  |  | | 947.4895 | | y8 |
|  | |  |  |  | |  |  | | 846.4419 | | y7 |
|  | |  |  |  | |  |  | | 745.3942 | | y6 |
|  | |  |  |  | |  |  | | 632.3101 | | y5 |
|  | |  |  |  | |  |  | | 472.2795 | | y4 |
| **Cashmere** |  | |  |  | |  |  |  | | |  |
| (DI)GLLDSEDC(CAM)K(DI)LPC(CAM)NPC(CAM)ATTNAYGK(DI) | | GLLDSEDCKLPCNPCATTNAYG K | 3+ | 890.0854 | | Light | 53 | | 1481.6512 | | y13 |
|  | |  |  |  | |  |  | | 782.4043 | | y7 |
|  | |  |  |  | |  |  | | 580.3089 | | y5 |
|  | |  |  |  | |  |  | | 395.2289 | | y3 |
|  | |  |  |  | |  |  | | 1074.5136 | | b9 |
|  | |  |  |  | |  |  | | 1187.5977 | | b10 |
|  | |  |  | 894.1105 | | Intermediate | 53 | | 1485.6763 | | y13 |
|  | |  |  |  | |  |  | | 786.4294 | | y7 |
|  | |  |  |  | |  |  | | 584.3340 | | y5 |
|  | |  |  |  | |  |  | | 399.2540 | | y3 |
|  | |  |  |  | |  |  | | 1082.5638 | | b9 |
|  | |  |  |  | |  |  | | 1195.6479 | | b10 |
|  | |  |  | 898.1297 | | Heavy | 53 | | 1489.6955 | | y13 |
|  | |  |  |  | |  |  | | 790.4487 | | y7 |
|  | |  |  |  | |  |  | | 588.3533 | | y5 |
|  | |  |  |  | |  |  | | 403.2733 | | y3 |
|  | |  |  |  | |  |  | | 1090.6023 | | b9 |
|  | |  |  |  | |  |  | | 1203.6864 | | b10 |
|  | |  | 4+ | 667.8158 | | Light | 40 | | 853.4414 | | y8 |
|  | |  |  |  | |  |  | | 782.4043 | | y7 |
|  | |  |  |  | |  |  | | 580.3089 | | y5 |
|  | |  |  |  | |  |  | | 395.2289 | | y3 |
|  | |  |  | 670.8347 | | Intermediate | 41 | | 857.4665 | | y8 |
|  | |  |  |  | |  |  | | 786.4294 | | y7 |
|  | |  |  |  | |  |  | | 584.3340 | | y5 |
|  | |  |  |  | |  |  | | 399.2540 | | y3 |
|  | |  |  | 673.8491 | | Heavy | 41 | | 861.4858 | | y8 |
|  | |  |  |  | |  |  | | 790.4487 | | y7 |
|  | |  |  |  | |  |  | | 588.3533 | | y5 |
|  | |  |  |  | |  |  | | 403.2733 | | y3 |
| (DI)GLGYGYGSSYGLGGYGGYGYGY FHPSFYGR | | GLGYGYGSSYGLGGYGGYGY GYFHPSFYGR | 3+ | 1069.8139 | | Light | 57 | | 476.2504 | | b5 |
|  | |  |  |  | |  |  | | 863.4159 | | y7 |
|  | |  |  |  | |  |  | | 726.3570 | | y6 |
|  | |  |  | 1071.1556 | | Intermediate | 57 | | 480.2755 | | b5 |
|  | |  |  |  | |  |  | | 863.4159 | | y7 |
|  | |  |  |  | |  |  | | 726.3570 | | y6 |
|  | |  |  | 1072.4954 | | Heavy | 57 | | 484.2947 | | b5 |
|  | |  |  |  | |  |  | | 863.4159 | | y7 |
|  | |  |  |  | |  |  | | 726.3570 | | y6 |
|  | |  | 4+ | 802.6122 | | Light | 42 | | 419.2289 | | b4 |
|  | |  |  |  | |  |  | | 476.2504 | | b5 |
|  | |  |  |  | |  |  | | 639.3137 | | b6 |
|  | |  |  |  | |  |  | | 696.3352 | | b7 |
|  | |  |  |  | |  |  | | 1230.5691 | | y10 |
|  | |  |  |  | |  |  | | 1010.4843 | | y8 |
|  | |  |  |  | |  |  | | 863.4159 | | y7 |
|  | |  |  |  | |  |  | | 726.3570 | | y6 |
|  | |  |  | 803.6185 | | Intermediate | 42 | | 423.2540 | | b4 |
|  | |  |  |  | |  |  | | 480.2755 | | b5 |
|  | |  |  |  | |  |  | | 643.3388 | | b6 |
|  | |  |  |  | |  |  | | 700.3603 | | b7 |
|  | |  |  |  | |  |  | | 1230.5691 | | y10 |
|  | |  |  |  | |  |  | | 1010.4843 | | y8 |
|  | |  |  |  | |  |  | | 863.4159 | | y7 |
|  | |  |  |  | |  |  | | 726.3570 | | y6 |
|  | |  |  | 804.6233 | | Heavy | 42 | | 427.2733 | | b4 |
|  | |  |  |  | |  |  | | 484.2947 | | b5 |
|  | |  |  |  | |  |  | | 647.3581 | | b6 |
|  | |  |  |  | |  |  | | 704.3795 | | b7 |
|  | |  |  |  | |  |  | | 1230.5691 | | y10 |
|  | |  |  |  | |  |  | | 1010.4843 | | y8 |
|  | |  |  |  | |  |  | | 863.4159 | | y7 |
|  | |  |  |  | |  |  | | 726.3570 | | y6 |
| **Yak** |  | |  |  | |  |  |  | | |  |
| (DI)GGVTC(CAM)GGLTYSTTAGR | | GGVTCGGLTYSTTAGR | 2+ | 793.3856 | | Light | 39 | | 856.4159 | | y8 |
|  | |  |  |  | |  |  | | 1083.5429 | | y11 |
|  | |  |  |  | |  |  | | 1243.5736 | | y12 |
|  | |  |  |  | |  |  | | 617.2712 | | b7 |
|  | |  |  | 795.3981 | | Intermediate | 39 | | 856.4159 | | y8 |
|  | |  |  |  | |  |  | | 1083.5429 | | y11 |
|  | |  |  |  | |  |  | | 1243.5736 | | y12 |
|  | |  |  |  | |  |  | | 621.2963 | | b7 |
|  | |  |  | 797.4078 | | Heavy | 39 | | 856.4159 | | y8 |
|  | |  |  |  | |  |  | | 1083.5429 | | y11 |
|  | |  |  |  | |  |  | | 1243.5736 | | y12 |
|  | |  |  |  | |  |  | | 625.3155 | | b7 |
|  | |  | 3+ | 529.2595 | | Light | 30 | | 592.3049 | | y6 |
|  | |  |  |  | |  |  | | 755.3682 | | y7 |
|  | |  |  |  | |  |  | | 856.4159 | | y8 |
|  | |  |  |  | |  |  | | 617.2712 | | b7 |
|  | |  |  | 530.6012 | | Intermediate | 30 | | 592.3049 | | y6 |
|  | |  |  |  | |  |  | | 755.3682 | | y7 |
|  | |  |  |  | |  |  | | 856.4159 | | y8 |
|  | |  |  |  | |  |  | | 621.2963 | | b7 |
|  | |  |  | 531.9409 | | Heavy | 30 | | 592.3049 | | y6 |
|  | |  |  |  | |  |  | | 755.3682 | | y7 |
|  | |  |  |  | |  |  | | 856.4159 | | y8 |
|  | |  |  |  | |  |  | | 625.3155 | | b7 |
| (DI)QC(CAM)C(CAM)ESNLEPLFNGYIET LR | | QCCESNLEPLFNGYIETLR | 2+ | 1186.0565 | | Light | 56 | | 1322.7103 | | y11 |
|  | |  |  |  | |  |  | | 1451.7529 | | y12 |
|  | |  |  |  | |  |  | | 920.3601 | | b7 |
|  | |  |  |  | |  |  | | 1049.4027 | | b8 |
|  | |  |  | 1188.0691 | | Intermediate | 56 | | 1322.7103 | | y11 |
|  | |  |  |  | |  |  | | 1451.7529 | | y12 |
|  | |  |  |  | |  |  | | 924.3852 | | b7 |
|  | |  |  |  | |  |  | | 1053.4278 | | b8 |
|  | |  |  | 1190.0786 | | Heavy | 56 | | 1322.7103 | | y11 |
|  | |  |  |  | |  |  | | 1451.7529 | | y12 |
|  | |  |  |  | |  |  | | 928.4044 | | b7 |
|  | |  |  |  | |  |  | | 1057.4470 | | b8 |
|  | |  | 3+ | 791.0401 | | Light | 43 | | 965.5051 | | y8 |
|  | |  |  |  | |  |  | | 1112.5735 | | y9 |
|  | |  |  |  | |  |  | | 1322.7103 | | y11 |
|  | |  |  |  | |  |  | | 1049.4027 | | b8 |
|  | |  |  | 792.3818 | | Intermediate | 43 | | 965.5051 | | y8 |
|  | |  |  |  | |  |  | | 1112.5735 | | y9 |
|  | |  |  |  | |  |  | | 1322.7103 | | y11 |
|  | |  |  |  | |  |  | | 1053.4278 | | b8 |
|  | |  |  | 793.7216 | | Heavy | 43 | | 965.5051 | | y8 |
|  | |  |  |  | |  |  | | 1112.5735 | | y9 |
|  | |  |  |  | |  |  | | 1322.7103 | | y11 |
|  | |  |  |  | |  |  | | 1057.4470 | | b8 |
| (DI)SISTVC(CAM)QPVGGVSTIC(CAM)QP AC(CAM)GVSR | | SISTVCQPVGGVSTICQPACGV SR | 3+ | 850.0821 | | Light | 46 | | 746.3614 | | y7 |
|  | |  |  |  | |  |  | | 1034.4506 | | y9 |
|  | |  |  |  | |  |  | | 1335.6144 | | y12 |
|  | |  |  |  | |  |  | | 804.3920 | | b7 |
|  | |  |  | 851.4238 | | Intermediate | 46 | | 746.3614 | | y7 |
|  | |  |  |  | |  |  | | 1034.4506 | | y9 |
|  | |  |  |  | |  |  | | 1335.6144 | | y12 |
|  | |  |  |  | |  |  | | 808.4171 | | b7 |
|  | |  |  | 852.7635 | | Heavy | 46 | | 746.3614 | | y7 |
|  | |  |  |  | |  |  | | 1034.4506 | | y9 |
|  | |  |  |  | |  |  | | 1335.6144 | | y12 |
|  | |  |  |  | |  |  | | 812.4364 | | b7 |
| (DI)YGC(CAM)GYGSGYSSYWPVC(CAM) YR | | YGCGYGSGYSSYWPVCYR | 2+ | 1105.4590 | | Light | 53 | | 694.3341 | | y5 |
|  | |  |  |  | |  |  | | 1217.5408 | | y9 |
|  | |  |  |  | |  |  | | 993.3771 | | b9 |
|  | |  |  | 1107.4715 | | Intermediate | 53 | | 694.3341 | | y5 |
|  | |  |  |  | |  |  | | 1217.5408 | | y9 |
|  | |  |  |  | |  |  | | 997.4022 | | b9 |
|  | |  |  | 1109.4812 | | Heavy | 53 | | 694.3341 | | y5 |
|  | |  |  |  | |  |  | | 1217.5408 | | y9 |
|  | |  |  |  | |  |  | | 1001.4215 | | b9 |
|  | |  | 3+ | 737.3084 | | Light | 40 | | 694.3341 | | y5 |
|  | |  |  |  | |  |  | | 880.4134 | | y6 |
|  | |  |  |  | |  |  | | 830.3138 | | b8 |
|  | |  |  | 738.6501 | | Intermediate | 40 | | 694.3341 | | y5 |
|  | |  |  |  | |  |  | | 880.4134 | | y6 |
|  | |  |  |  | |  |  | | 834.3389 | | b8 |
|  | |  |  | 739.9898 | | Heavy | 40 | | 694.3341 | | y5 |
|  | |  |  |  | |  |  | | 880.4134 | | y6 |
|  | |  |  |  | |  |  | | 838.3581 | | b8 |

Note: The modified peptide form with dimethyl labels (DI) and carbamidomethyl modification (CAM) as well as the original peptide sequence are listed separately. Peptides from the reference were labeled with the light dimethyl tag and peptides from replicates of a given fiber sample were labeled with the intermediate or heavy dimethyl tags.
