# Supplementary material for: Combining Untargeted and Targeted Proteomic Strategies for Discrimination and Quantification of Cashmere Fibers
Source: PLoS One. 2016 Jan 20;11(1):e0147044. doi: 10.1371/journal.pone.0147044 (PMC4720366; doi:10.1371/journal.pone.0147044)
Supplement: S4 Table — (DOC) [file pone.0147044.s005.doc]

**Supporting Information for Manuscript**

**S4 Table. PRM identification results of 10 peptide markers in QC samples and textile fabrics**

| **Peptide markers** | **QC1***^a^* | **QC2** | **QC3** | **QC4** | **QC5** | **T1*^b^*** | **T2** | **T3** | **T4** |
| --- | --- | --- | --- | --- | --- | --- | --- | --- | --- |
| W-1 | +*^c^* | + | + | + | + | -*^c^* | + | + | - |
| W-2 | + | + | + | + | + | + | + | + | + |
| W-3 | + | + | + | + | + | - | - | - | - |
| W-4 | + | + | + | + | + | + | + | + | + |
| C-1 | + | + | + | + | + | + | + | + | + |
| C-2 | + | + | + | + | + | + | + | + | + |
| Y-1 | - | - | - | + | + | - | - | - | - |
| Y-2 | - | - | - | + | + | - | - | - | - |
| Y-3 | - | - | - | + | + | - | - | - | - |
| Y-4 | - | - | - | + | + | - | - | - | - |

*^a^* QC1, a mixture of W1 and C1 (2:3, *w/w*); QC2, a mixture of W1 and C1 (3:2, *w/w*); QC3, a mixture of W1 and C1 (4:1, *w/w*); QC4, a mixture of W1, C1 and Y (1:1:1, *w/w/w*); QC5, a mixture of W1, C1 and Y (1:3:1, *w/w/w*).

*^b^*T1~T4 refer to the four fabric samples evaluated in this study.

*^c^*“+” stands for detected; “-” stands for undetected.
